# Impact Resistant Structure Design and Optimization Inspired by Turtle Carapace

**DOI:** 10.3390/ma15082899

**Published:** 2022-04-15

**Authors:** Baoqing Pei, Lei Guo, Xueqing Wu, Mengyuan Hu, Shuqin Wu, Yangwei Wang

**Affiliations:** 1 Beijing Key Laboratory for Design and Evaluation Technology of Advanced Implantable & Interventional Medical Devices, Beijing Advanced Innovation Center for Biomedical Engineering, School of Biological Science and Medical Engineering, Beihang University, Beijing 100083, China; pbq@buaa.edu.cn (B.P.); guolei10@buaa.edu.cn (L.G.); by2110106@buaa.edu.cn (M.H.); 2 School of Big Data and Information, Shanxi College of Technology, Shuozhou 036000, China; 3 National Key Laboratory of Science and Technology on Materials under Shock and Impact, Beijing Institute of Technology, Beijing 100081, China; wangyangwei@bit.edu.cn

**Keywords:** bionic structure, turtle carapace, sandwich structure, impact resistance

## Abstract

The turtle carapace has a high level of protection, due to its unique biological structure, and there is great potential to use the turtle carapace structure to improve the impact resistance of composite materials using bionic theory. In this paper, the chemical elements of the turtle carapace structure, as well as its mechanical properties, were investigated by studying the composition of the compounds in each part. In addition, the bionic sandwich structure, composed of the plate, core, and backplate, was designed using modeling software based on the microstructure of the keratin scutes, spongy bone, and the spine of the turtle carapace. Additionally, finite element analysis and drop-weight experiments were utilized to validate the impact-resistant performance of the bionic structures. The numerical results show that all of the bionic structures had improved impact resistance to varying degrees when compared with the control group. The experimental results show that the split plate, the core with changing pore gradients, and the backplate with stiffener all have a considerable effect on the impact-resistance performance of overall composite structures. This preliminary study provides theoretical support for composite material optimization.

## 1. Introduction

As armed helicopters enjoy the advantages of high mobility and high attack firepower, they are an efficient anti-tank weapon in modern military conflicts. Enhancing the protection capability of armed helicopters is of great importance to enhancing the air control of military forces on the battlefield. However, with the increasing difficulty and cost of developing new protective materials, attention has been focused on structural optimization as a way to improve the impact resistance of protective materials [1,2]. Many organisms in nature have efficient protective armor, and studying the microstructure of their armor is arguably the simplest method to find a structure that offers effective impact resistance using bionic theory [3,4,5].

Bionic structures are already used in many industries. For example, many thin-shelled, arch-shaped bionic structures are widely used in the construction industry [6]. Many biological microstructures have also been investigated, with Yang et al. [7] developing a heat sink to improve the heat dissipation efficiency of batteries by mimicking the microstructure of shark skin. Feng et al. [8] drew on bionic ideas to optimize the composite using a bionic double-gradient porous structure, which increased the effective thermal conductivity of the material by 226%. Chang et al. [9] significantly improved the sensitivity of the sensor through the optimization of the internal structure by imitating the multilayer structure of the reed leaf surface. However, there is less information available about the application of bionic structures in the field of impact.

Turtles are slow-moving and weak in terms of attack capabilities, yet they have survived because they have evolved hard carapace armor to defend themselves from predators. If the excellent structure of the turtle carapace can be applied to the optimization of protective armor, it may be possible to improve the impact resistance of protective armor. Research has been carried out on the mechanical properties of turtle carapaces, and such studies have attracted the attention of many scholars [10,11]. For example, Damiens et al. [12] analyzed American box turtle carapaces in dry conditions by compression experimental experiments and finite element simulations, which showed a trinal curve at each compression rate, typical of the quasi-static compression behavior of sandwich core structures. Achrai et al. [13] demonstrated mechanically that a turtle carapace containing turtle carapace keratin scutes had a much better performance than a turtle carapace without keratin scutes. Microscopic observation revealed that the whole shells is formed by the stacking of closely arranged flakes. Based on the enhanced energy absorption mechanism of turtle carapace surface keratin scutes, Achrai et al. [14] prepared the first impact-resistant structure imitating turtle carapace armor by coating a ductile polymer film onto a brittle material, and found that the energy absorption capacity of the structure was significantly enhanced under certain conditions through a series of impact experiments.

Multiple material properties combined with composite structures can have surprising effects. Trukhanov et al. [15] found that the use of carbon nanoforms in composite materials could improve the mechanical properties. At the same time, if oxygen-based composite materials are used, they can be applied to radar technologies in aircraft [16]. Han et al. [17] likened the titanium–aluminum (Ti-Al) laminate structure to the keratin scutes of turtle carapace and the SiC fiber-reinforced titanium matrix composite to the outer dense bone layer of turtle carapace. Experimental results show that the bionic structure combines the high toughness of titanium metal with the high strength of SiC fiber-reinforced titanium matrix composites and is an imitation of the turtle carapace structure, with excellent mechanical properties. Lightweight sandwich structures are used extensively in aerospace due to their excellent energy absorption capability and high flexural stiffness-to-weight ratio [18]. At present, research on sandwich structures mainly focuses on the optimization of their core structures [18]. For example, Wang et al. [19] conducted a blast experiment, and it was found that the foam structure with pore variation has a better energy absorption capacity. According to Jing et al. [20], the blast resistance and energy absorption capabilities of sandwich panels with layered gradient metallic foam cores could be improved. Huang et al. [21] found by experiment with a gas gun that the foam-filled lattice structure had better impact resistance compared with the truss core structure only. However, there are few reports on the optimization of plate and backplate [22,23,24,25]. As a result, the structural optimization of the plate and backplate has a high potential for improving the sandwich structure’s impact resistance.

Even though considerable research has been carried out on the turtle carapace, most of which focuses on the microstructure and biomechanical properties of the turtle carapace, research on the chemical composition within the turtle carapace, the resistance to the impact of the overall bionic composite structure, and the enhancement mechanism of the synergistic effect between the layers has rarely been reported. Therefore, this paper aims to investigate the chemical composition of the internal elements of the turtle carapace and study the impact resistance of the multi-layered hierarchical structure of the turtle carapace and the synergistic enhancement mechanism between the layers through finite element simulations and mechanical experiments, thus providing theoretical and technical support for the subsequent optimization of aerospace protective armor.

## 2. Materials and Methods

The research was approved by the Science and Ethics Committee of the School of Biological Science and Medical Engineering in Beihang University (protocol code: BM201900125; date of approval: 26 February 2019).

### 2.1. Elemental Analysis of Turtle Carapace

An adult red-eared turtle, 24–25 cm long and weighing 2.5 kg, was selected as the sample, dissected at room temperature, and then air-dried, cut, and polished. An energy-dispersive spectrometer (EDAX, FEI Genesis, 2000) was employed to analyze specific areas of the turtle carapace substratum for spot elements in the keratin scutes, the layer of spongy bone, the attachment area of the adjacent turtle carapace, and the attachment area between the cuticle and the bone layer.

### 2.2. Structural Design

The structure of the turtle carapace is a typical sandwich structure, with the bone layers of the turtle carapace consisting of the upper dense layer, the middle spongy bone layer, and the lower dense layer, corresponding to the plate, core, and backplate of the sandwich structure, respectively. Based on a previous study of the carapace of the adult red-eared turtle, the thickness ratio of the three layers is approximately 1:4:1. The total thickness of the sandwich composite structures designed here is 30 mm, a value derived from our previous research on armor. Hence, the plate, core, and backplate thicknesses are 5 mm, 20 mm, and 5 mm, respectively. The length and width are both 50 mm. A monolithic block plate with a typical sandwich structure, a core with a constant porous structure, and a backplate with a typical orthogonal stiffener were used as control groups.

**Figure 1:**
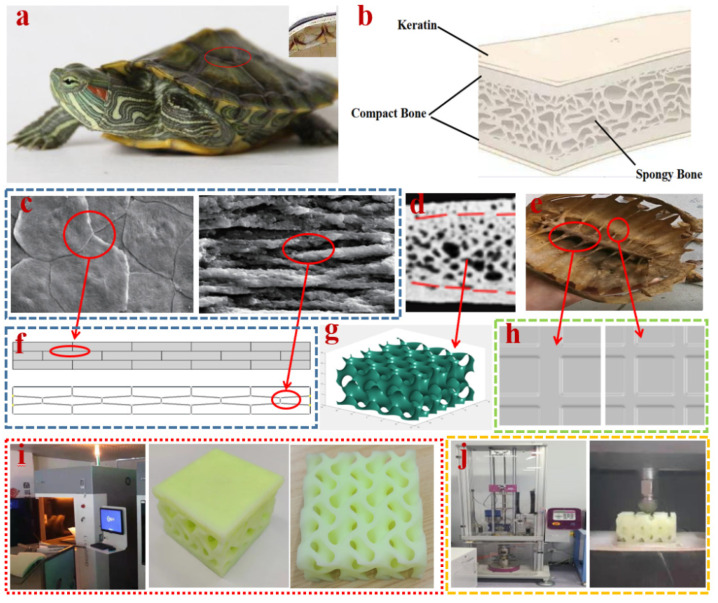
Bionic schematic diagram of a turtle carapace. (**a**) The partial enlargement of the turtle carapace. (**b**) The microscopic structure of the tortoise carapace. (**c**) The microstructure of keratin [29]. (**d**) The microstructure of the spongy bone region. (**e**) The turtle’s spine. (**f**) Plate. (**g**) Core. Structure from MATLAB. (**h**) Backplate structure. (**i**) The 3D printed composite structural specimens. (**j**) Drop-weight impact experiment.

Due to the excellent mechanical properties of keratin scutes, the microstructure of the dense layer of turtle carapace consists of a stack of sheets [14,26,27]. The microstructure of the keratin scutes of the turtle carapace is shown in Figure 1c. The two plate structures shown in Figure 1f were designed based on this microstructure, being composed of three layers of square and rhombus parts stacked alternately. The core structures were designed according to Figure 1d, and a typical cube structure was used as the control group of plates. The three plate structures mentioned above have the same volume and mass. The porous structures were generated by MATLAB’s meshgrid function combined with the G minimal surface function (as shown in Equation (1)), under the condition that the pore size was guaranteed to be the same as the control group of the core [28].

(1) F(x,y,z)=sin(X)cos(Y)+sin(Z)cos(X)+sin(Y)cos(Z)+t

The volume parameter “*t*” is adjusted to produce porous structures with variable pore gradients [30], as shown in Figure 1g. The porous gradient structures are placed forward and backward, respectively, where the forward placement is a negative pore gradient (pore size gradually decreasing from plate to backplate), named Core B, and the backward placement is named Core C. These can be considered two types of core structures. Based on the coupling of the turtle carapace back to the spine, two stiffeners structure (orthogonal) as the control group of backplate. Backplate B and Backplate C were designed to ensure the same total volume of the backplate, as shown in Figure 1h. The volume, thickness, and area of the stiffeners of the three backplate structures are the same. The structure designed in this paper is shown in Table 1.

### 2.3. Finite Element Simulations

In this paper, the object chosen to simulate a 12.7 mm-diameter and 155 mm-long military projectile is a conical object [31], which is simulated into the perpendicular direction to the plate for high-speed impact. The material property of the conical object is set to steel, and the deformation does not need to be considered, so it is set to rigid body [32]. Designed structures were discretized into the tetrahedral (C3D4) element type and the hexahedral (C3D8) element type. A mesh convergence test was conducted on the intact model under an impact velocity of 100 m/s.

The composite structures were set to a tie constraint between the layers. The shock equation of state (linear) was used in the computational process [33,34]. The contact type between the impact object and the composite structures was set to surface-to-surface contact, with the impact velocity of 100 m/s. The contact algorithm was penalty, which was set as 0.3. The time was set as 0.01 s, and the incrementation was set as the type of automation. The location of impact is the center point of the outer surface of the plate. The four edges of the models are set with fixed constraints during the calculation. Firstly, all the plates, cores, and backplates are individually simulated to verify the impact resistance of the bionic structure compared with the control groups. A total of 27 composite structures were then assembled in the order plate–core–backplate, and finite element simulations were then carried out to verify the impact resistance of the bionic composite structures. The resistance to impact was measured by the amount of change in kinetic energy before and after the conical object penetrated the composite structure. The material property chosen for the finite element simulations was aluminum (Al7075-T651) [35]. The model chosen for damage evolution due to impact was Johnson–Cook, as shown in Equation (2) [36,37].

(2) $$\sigma =(A+B\varepsilon^{n})[1+C\ln({\dot{\varepsilon }}^{*})][1-T^{*}{}^{m}]$$

where σ is the equivalent flow stress; ${\dot{\varepsilon }}^{*}$ is the dimensionless strain rate $\left[{\dot{\varepsilon }}^{*}=\dot{\varepsilon }/{\dot{\varepsilon }}_{0}\right]$, where $\dot{\varepsilon }$ is the equivalent plastic strain, and ${\dot{\varepsilon }}_{0}$ is the reference strain rate; $T^{*}$ can be calculated through $T^{*}=(T-T_{ref})/(T_{m}-T_{ref})$, where $T_{ref}$ is the reference deformation temperature and $T_{m}$ is the melting temperature of the material; *A* is the initial yield strength; *B* is the strain-hardening coefficient; *n* is the strain-hardening exponent; *C* is the strain rate sensitivity; *n* is the strain-hardening exponent; and *m* is the thermal-softening exponent. The Johnson–Cook model parameters for Al 7075-T651 are listed in Table 2.

Johnson–Cook proposed a failure model using strain rate and temperature, which has the damage parameter *D* [36]:

(3) $$D=\sum_{t=0}\Delta \varepsilon_{pl}/\dot{\varepsilon }$$

where $\Delta \varepsilon_{pl}$ is the variation of the equivalent plastic strain. ${\bar{\varepsilon }}_{f}^{pl}$ depends on stress triaxiality, strain rate and temperature, and it can be defined as:

(4) $${\bar{\varepsilon }}_{f}^{pl}=[D_{1}+D_{2}e^{D_{3}\sigma^{*}}](1+D_{4}\ln({\dot{\varepsilon }}^{*}))(1+D_{5}T^{*})$$

where $D_{1}$, $D_{3}$, $D_{4}$ and $D_{5}$ are the damage parameters of the material under consideration. The Johnson–Cook model failure parameters for Al 7075-T651 are listed in Table 3.

### 2.4. Drop-Weight Experiments

The six composite structures B-B-A, B-B-B, B-B-C, B-C-A, B-C-B, and B-C-C were selected for 3D printing based on the numerical results, and the printing material was resin. The properties of the resin are shown in Table 4. The drop-weight experiment was conducted to verify the impact resistance of the composite structures. As shown in Figure 1j, the Instron CEAST 9340 drop-weight experimental machine was used for the drop-weight experiment. The drop-weight loading mass was 5 kg with a speed of 4 m/s. To prevent damage to the experimental equipment due to excessive impact energy, the full penetration of the specimen was not performed. The data collected in the experiments were the depth of depression caused by the drop-weight machine on the specimen and the kinetic energy absorbed by the specimen during the impact. The depth of depression was used to evaluate the impact resistance of the specimen.

## 3. Results

### 3.1. Elemental Analysis of Turtle Carapace

**Figure 2:**
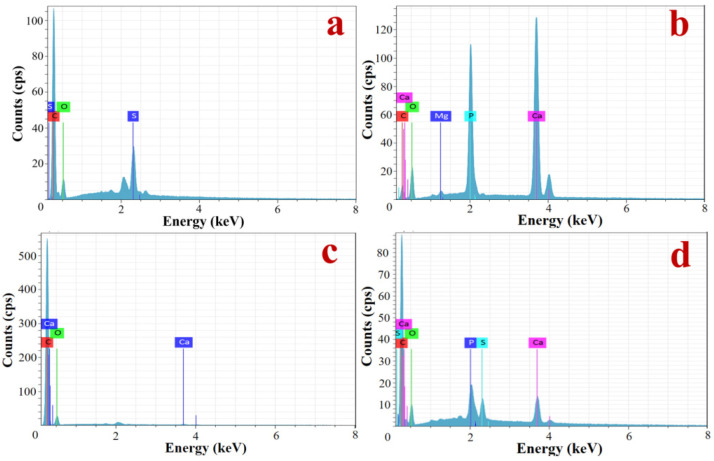
Element detection diagram of the turtle carapace. (**a**) Cuticle layer. (**b**) Bone layer. (**c**) Connection area. (**d**) The junction between the keratin scutes and the bone.

The keratin scutes consist of keratin, which is a fibrillated hard keratin, and contain a large amount of β-keratin, which is a β-sheet structure; the distribution of elements in keratin scutes is shown in Figure 2a, with C, O, and S as the main elements, of which S has a particularly high content. The results of elemental testing on the bone layer are shown in Figure 2b. Similar to the bone structure of other spinal organisms, the bone contains quantitative amounts of mineral elements such as Ca, Mg, and P, with Ca and P in high amounts. The mineral elements are mainly the constituent elements of bone salts. The bone contains organic and inorganic components, of which the former determines its elasticity, and the latter determines its hardness and stiffness. The results of the adjacent turtle carapace connection area are shown in Figure 2c, which contains C, O, and a small amount of Ca, with low mineralization and a large number of collagen fibers at the joint. The elements of the junction area between the keratin scutes and the bone layer were examined, as shown in Figure 2d. The junction contains C, O, S, Ca, and P, and contains all the elements of the keratin scutes and the bone layer, but the content of S is lower than that of the keratin scutes, and the content of Ca and P is significantly lower than that of the bone layer, indicating that there is a clear transition zone between the keratin scutes and the bone layer.

### 3.2. Numerical Results

**Figure 3:**
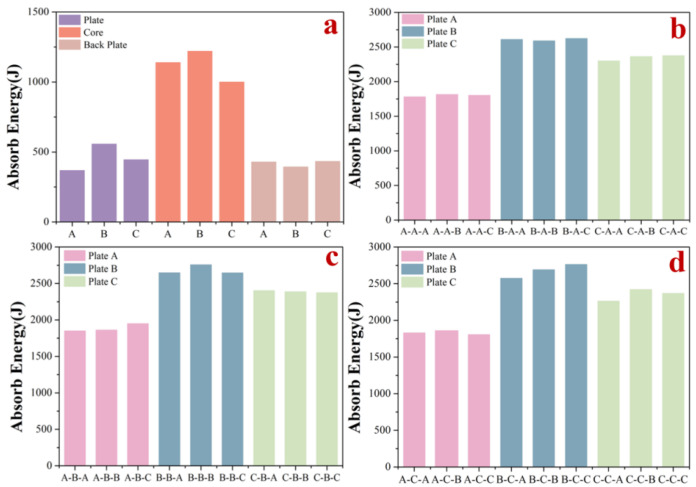
Numerical results. (**a**) Energy absorption statistics of impact resistance of nine components. (**b**) Energy absorption statistics of core A composite structure against impact. (**c**) Energy absorption statistics of Core B composite structure against impact. (**d**) Energy absorption statistics of Core C composite structure against impact.

In this paper, all the structures are divided into plate groups, core groups, and backplate groups, depending on their position in the composite structure. Finite element simulations of projectile impact were performed for all the plates, cores, and backplates. From the value of absorbed kinetic energy, the numerical results of the impact resistance of each group are shown in Figure 3a. Plate group: Plate B > Plate C > Plate A; core group: Core B > Core A > Core C; backplate group: Backplate C > Backplate A > Backplate B.

The damage to Plate B and Plate C was significantly greater than that to Plate A when each component was individually impacted. Compared with Plate A, both Plate B and Plate C showed larger bulges and more deformation after the impact, and more fragments were produced after the impact was completed. Core B had the best performance among the three structures in terms of resistance to impact. However, the difference in kinetic energy absorbed by the three structures during the process was small, and Core B and Core C were identical, but the difference in kinetic energy absorbed due to the different placement directions was 224 J. The impact resistance of Core B was 21.9% higher than that of Core C. Of the three backplate structures, Backplate C was not significantly more resistant to impact than Backplate A, while Backplate B showed a decrease compared with Backplate A. The backplate construction with its different stiffener arrangements did not show a significant influence on the improvement of the anti-impact capacity.

**Figure 4:**
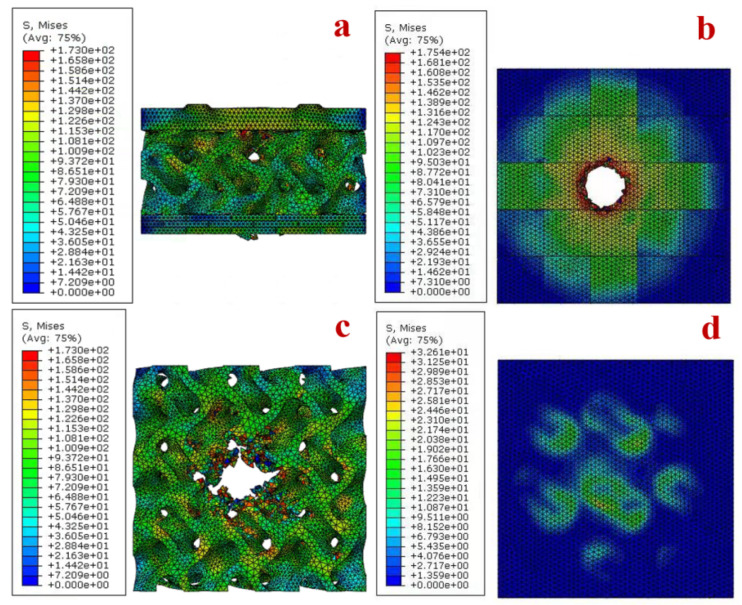
The stress cloud diagram of the B-B-C composite structure and its components. (**a**) B-C-C composite structure. (**b**) Plate B. (**c**) Core C. (**d**) Backplate C.

Numerical results are shown in Figure 3b–d, where all composite structures were completely penetrated by the intruding objects. The A-A-A composite structure was the weakest of the 27 composite structures in terms of impact resistance. The B-C-C and B-B-B composite structures were the two structures with the highest impact resistance among the 27 composite structures, of which the stress cloud of the B-C-C composite structure and its constituent parts are shown in Figure 4. The kinetic energy absorbed by all the composite structures was found to be greater than the sum of the kinetic energies absorbed by their constituent parts when subjected to individual impact. In the case of the B-B-C composite structure, for example, large plate bulges and deformations occurred when the plate was impacted, and damage was caused to the constituent units of the plate beyond the point of impact due to compression by neighboring units. Composite structures fitted with Core B were consistently more resistant to impact than those fitted with Core A, while most composite structures fitted with Core C were more resistant to impact than those fitted with Core A. The layout of the backplate stiffeners had no significant influence on the overall composite structure’s resistance to impact.

### 3.3. Drop-Weight Impact Experiment

**Figure 5:**
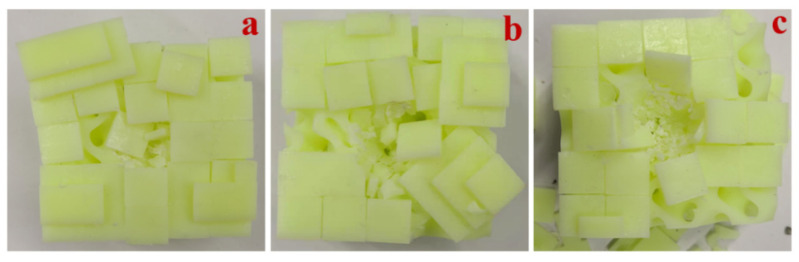
Experimental results. (**a**) B-C-A composite structure. (**b**) B-C-B composite structure. (**c**) B-C-C composite structure.

**Figure 6:**
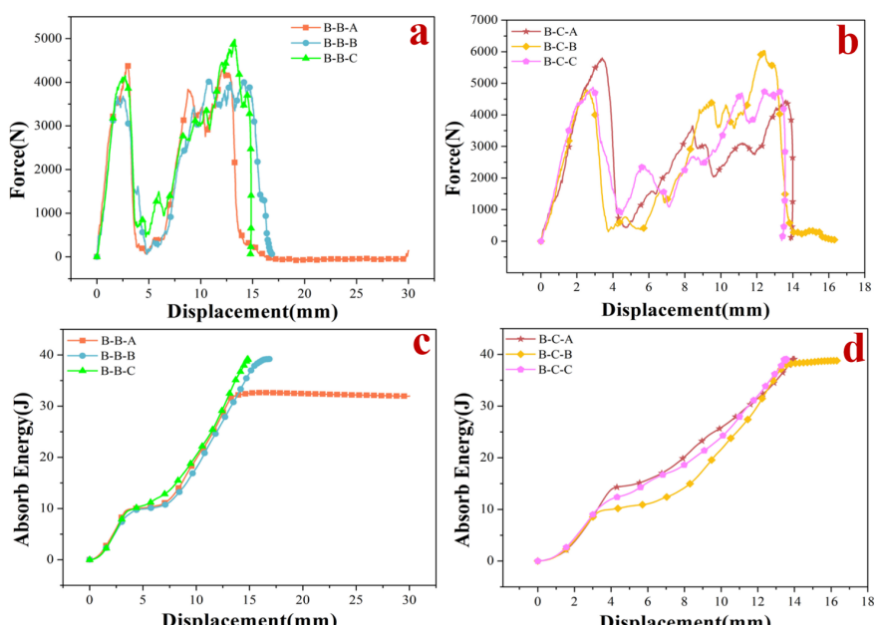
(**a**) Core B group stress–displacement curve. (**b**) Core C group stress–displacement curve. (**c**) Core B group absorbed energy–displacement curve. (**d**) Core C group absorbed energy–displacement curve.

The numerical results show that plate B was more resistant to impact than the other two plates, and both core B and core C exhibited greater impact resistance in the composite structures. Six composite structures, namely B-B-A, B-B-B, B-B-C, B-C-A, B-C-B, and B-C-C, were selected for 3D printing. Since the drop-weight experiment did not completely penetrate the specimen, the experiments were divided into two groups according to the difference in the cores. For example, in terms of the core C group, after the impact of the specimens, the plate produced a large bulge, as can be seen in Figure 5. This phenomenon is the same as the numerical result. Among them, the B-C-B composite structure appeared to have a greater degree of damage, and the components of the panel were partially falling off. The data obtained from the experiments were fitted into the graph shown in Figure 6. Among them, the B-B-A composite structure was completely penetrated, and large cracks appeared in the core of the B-C-A composite structure. The remaining composite structures had only some of the constituent parts of the plates detached, followed by depressions of varying depths in the cores. When subjected to the impact, the B-C-C composite structure specimens showed the smallest depression depth of 13.799 mm of all of the above composite structures. The B-B-C composite structure showed the smallest depression depth of the composite structures of the Core B group.

## 4. Discussion

This paper proposes the optimization of the structures of protective materials using the structure of a bionic turtle to improve the resistance to impact, because of the increasing difficulties in developing new materials and rising costs. Microstructural observations show that a multilayer structure of keratin scutes is formed by the accumulation of keratin in a parallel manner, which exhibits a high degree of toughness and tensile strength. Furthermore, the large number of S elements represents a high content of cysteine in the keratin scutes, which can be chemically reacted to form keratin suites with a certain degree of stiffness and rigidity [38]. The presence of fibers at the joints of the adjacent turtle carapace increases mobility and provides a degree of cushioning in the event of an impact. The presence of a connecting region between the keratin scutes and the bone layer tissue contributes to a perfect connection between the two, making the overall structure of the turtle carapace stronger, but the mechanical properties of this region remain unclear. Therefore, the microstructure of the keratin scutes is applied to the plate to optimize the scutes to the upper dense bone layer instead.

The numerical results show that the amount of energy absorbed by the composite structure during the impact resistance process is greater than the sum of the energy absorbed by the components of the composite structure, so there must be a coordination relationship between the components of the composite structures to achieve the effect of 1 + 1 + 1 > 3. The stress cloud calculated by numerical results is shown in Figure 4b. When Plate A is penetrated, the stress is propagated outward in a circular shape from the contact point between the penetrated object and the plate. However, when Plate B and Plate C are penetrated, the stress propagates significantly faster than in the ring direction along the edge of the constituent units at the impact point, and this stress propagation mechanism may enhance the resistance of the bionic plate to impact. From Figure 3b–d, it can be seen that, when the core and backplate are the same, different plates have different influences on the impact resistance of the composite structure. Plate B and Plate C produce more fragments and a larger bulge on the ejection side of the plate than Plate A when subjected to impact. As shown in Figure 4c, the stress propagation rate of the core structure is significantly slower than that of the plate, which therefore increases the damage of the core structure during the impact. The role of the cores in the impact process is that when the bullet penetrates the plate, the plate will produce some fragmentation and the lower part of the plate will bulge. At this point, a small amount of bullet kinetic energy is transferred to the plate fragments. The fragments follow the bullet impact core, the bulge, and fragments on the plates of the joint action, which also increases the damage to the core. The porous structure has been widely used in protective devices, mainly because the mechanism of the porous structure against impact is mainly through its plastic deformation to absorb kinetic energy, and the greater the degree of deformation, the more kinetic energy is absorbed [39,40,41]. Additionally, the change in pore gradient of the core affects the resistance to impact, and the structure with a negative gradient is more suitable for impact resistance applications, which is consistent with the results of Ajdari on gradient honeycomb structures [42,43].

From the numerical statistics of the absorbed kinetic energy of the 27 composite structures, it can be seen that there is no clear pattern in the effect of the stiffener layout of the backplate on the composite structure, but the different layouts have different influences on the impact resistance of the composite structure. It can be observed from Figure 4d that the backplate mainly supports the core before direct contact with the intruding object, and the backplate resists the impact of the intruding object with its strength once the core is fully penetrated. In this experiment, composite structures with different cores perform differently when the plate and backplate are the same, with the composite structures with Core B performing best in terms of impact resistance. Overall, the composite structures with Core C perform better than the composite structures with Core A. Therefore, cores with a positive gradient are more suitable for use in impact-resistant composite structures than porous structures with uniform pores.

As can be seen in Figure 5 and Figure 6, during the impact process, the more severely the components of the panel fall off, the less kinetic energy is absorbed. The more severely damaged the plate, the lower the energy absorption efficiency, so the stiffness and strength of the panel have a great influence on the impact resistance. The trend of all curve changes is essentially the same in each grouping; the core is the same in each group. The higher peak loads generated during plate penetration contribute to the lower peak loads during core impact, mainly because the hammerhead dissipates more kinetic energy during plate impact when generating higher peak loads. Therefore, increasing the hardness and strength of the plates will reduce the penetration depth of the core, thus increasing the overall structural resistance to impact. When the core is different, the composite structures fitted with Core C have a higher peak load compared with the composite structures fitted with Core B when the plate is penetrated. However, it does not mean that the composite structures with Core C are more resistant to impact than those with Core B, as none of the above experiments fully penetrated the composite structures, and the depth of depression caused by the impact is influenced by the density of the material. The composite structures with Backplate C are the most resistant to impact, and those with Backplate A are the least resistant, so the layout of the backplate stiffener has a significant effect on the impact resistance of the composite structures.

In summary, the B-C-C composite structure has the strongest impact resistance. If the hard-soft nanocomposites are synthesized by the in situ sol-gel route, the armor with a B-B-C composite structure could improve electromagnetic performance while ensuring strength, laying the foundation for future electronic warfare [44]. There were some limitations in the context of model settings and experiment design. Only the structure was considered, and not the impact resistance of materials and dimensions. For example, the equation of state for finite elements is only linear. Another important limitation is that to prevent excessive kinetic energy from causing damage to experimental equipment, there was only a partial correspondence between the experiment and the numerical calculation. We will continue to optimize experiments in the future, such as gas gun experiments, to make them more relevant.

## 5. Conclusions

This study aims to improve the impact resistance of existing aerospace armor by optimizing the use of bionic structures to address the light weight and high-speed impact resistance of aerospace protective armor. The effectiveness of the bionic composite structures designed in this paper is verified using finite element simulations and drop-weight experiments. The following main conclusions are drawn:

1. Cysteine, which contains the element S, chemically binds the keratin in the keratin scutes together, which has great mechanical properties.
2. The application of the turtle carapace to the optimization of armor with a typical sandwich composite structure and no variation in mass and volume will significantly increase the impact resistance, and may provide insight into the subsequent development of protective armor for aerospace applications.
3. The plate plays a crucial role in absorbing energy, and its increased stiffness and strength can reduce the depth of depression of the core to a greater extent. The use of negative-gradient structures can improve the energy absorption efficiency of the cores.
4. A backplate with a reasonable stiffener layout will improve the impact resistance of composite structures when they are not fully penetrated.

## Figures and Tables

**Table 1:** The structure designed in this paper.

Plate Group	Name	Core Group	Name	Backplate Group	Name
Control	Plate A	Control	Core A	Control	Backplate A
Square	Plate B	Negative Gradient	Core B	Three Stiffeners	Backplate B
Rhombus	Plate C	Positive Gradient	Core C	Four Stiffeners	Backplate C

**Table 2:** Johnson–Cook Strength Model [36].

Sr. No.	Property	Value	Unit
1	Strain Rate Correlation	First-Order	
2	Initial Yield Stress	835.833	MPa
3	Strain-hardening Coefficient	473.667	MPa
4	Strain-hardening Exponent	0.561	
5	Strain Rate Sensitivity	−0.08581	
6	Thermal Softening Exponent	4.2285	
7	Melting Temperature	873	K
8	Reference Strain Rate (/sec)	0.0005	

**Table 3:** Johnson–Cook failure model parameters for Al 7075-T651 [36].

Sr. No.	Property	Value
1	Damage Constant D1	0.1009
2	Damage Constant D2	0.1214
3	Damage Constant D3	−0.9150
4	Damage Constant D4	0.16789
5	Damage Constant D5	0.877675
6	Melting Temperature	873 K
7	Reference Strain Rate (/sec)	1

**Table 4:** The properties of resin.

Sr. No.	Property	Value	Unit
1	Hardness	85	Shore D
2	Flexural modulus	2692–2775	Mpa
3	Flexural strength	69–74	Mpa
4	Tensile modulus	2589–2695	Mpa
5	Tensile strength	38–56	Mpa
6	Elongation at break	8–12%	
7	Poisson’s	0.4–0.44	
8	Impact strength notched Izod	45–55	J/m
9	Heat deflection temperature	38~50	°C
10	Coefficient of thermal expansion	0.000097	°C

## Data Availability

The data are available upon request.
